# Running Economy Benefits of Shoes Incorporating Advanced Footwear Technology Decrease With Increasing Incline and Are Negligible Above Moderate Gradients

**DOI:** 10.1111/sms.70298

**Published:** 2026-05-12

**Authors:** Graham N. Askew, Sophia Hill, Molly Payne, Todd D. Stewart, Danielle Charles, Andy P. Brown

**Affiliations:** ^1^ School of Biomedical Sciences University of Leeds Leeds UK; ^2^ School of Mechanical Engineering University of Leeds Leeds UK; ^3^ School of Chemical and Process Engineering University of Leeds Leeds UK

**Keywords:** biomechanics, carbon fiber plate, elastic mechanism, energy cost, gradient, Nike Vaporfly shoe, super‐shoes

## Abstract

Shoes incorporating advanced footwear technology (AFT) improve running economy (RE) during level running by ~3%–4% compared to traditional shoes, but the extent to which benefits are preserved on inclines is unclear. Here we investigated the impact of AFT shoes on the energetics of incline running and examined how much benefits are reduced relative to level running. Twelve competitive male runners ran on a treadmill at four gradients (level, 3%, 6%, and 9%) in the Nike ZoomX Vaporfly Next% shoe (VF, AFT) and the Nike ZoomX Streakfly shoe (SF, control) in randomized order. Oxygen consumption was measured using indirect calorimetry, and shoe material properties determined using indentation and cantilever bending tests. The RE benefit of the VF over the SF decreased exponentially with gradient (*p* < 0.001; *R* ^2^ = 0.23) from 4.22% during level running, declining by ~20% per 1% increase in gradient, to 2.42% at 3% incline, 1.05% at 6% incline and 0.52% at 9% incline. The VF stored more energy under compression than the SF but had lower resilience. In bending, the VF was ~4‐fold stiffer than the SF (4.99 N/mm vs. 1.17 N/mm) with comparable resilience. The exponential decrease in RE benefit with increasing incline suggests that the performance advantage of the VF comes from the elastic properties of the midsole and carbon plate combined, contributing less as the relative importance of elastic mechanisms decreases at steeper inclines. The VF therefore provides the greatest performance benefit on flat courses, with smaller but still present gains on shallow inclines typical of undulating courses.

## 1 Introduction

Lightweight running shoes featuring a curved, midsole carbon fiber plate embedded in a relatively thick resilient polyether block amide (PEBA) mid‐sole foam, gained an upsurge in popularity following their high‐profile introduction during Eliud Kipchoge's 2017 attempt to run a marathon in under 2 h [1]. The emergence of such *advanced footwear technology* (AFT) shoes or ‘super’ *shoes* coincided with a spate of World record performances in long‐distance running, from 5 km to the marathon, across both women's and men's events [2], including the recent achievement of sub‐2‐h marathon times. For example, marathon finishing times improved by 2.0% in males and 2.6% in females from pre‐AFT to post‐AFT performances [2]. Nike first introduced AFT in a prototype version of the Vaporfly shoe in 2016, followed by the commercial release of the Vaporfly 4% shoe in 2017. This latter model has been shown to reduce the energetic cost of level running by approximately 3%–4% compared to a traditional marathon running shoe [3, 4, 5, 6], which is consistent with (though not proportional to) the improvements in performance times [3, 7]. Similar reductions in energetic cost (3%–4%) have been reported for several other models of AFT shoe (e.g., Asics Metaspeed Sky and Saucony Endorphin Speed 2 shoe), although not all models provide this magnitude of benefit [5, 8, 9].

Several biomechanical explanations for the energetic benefits of AFT have been proposed. The energetic cost of running increases by ~1% per 100 g mass per shoe [7, 10], thus the lightweight nature of AFT compared to traditional marathon shoes contributes to improved running economy (RE). However, RE improves in weight‐matched shoes, indicating that there must be other factors [4]. One such factor is the use of low‐density compliant and resilient PEBA foam in the midsole, which compresses and recoils during early stance, returning energy to the body's centre of mass during late stance [3]. Compared to conventional ethylene vinyl acetate (EVA) foams, PEBA foams improve RE by 1%–2% [11, 12]. Increased stack height further facilitates the storage and return of elastic strain energy, while also increasing effective leg length, a characteristic associated with improved RE [13]. Additionally, the embedded midsole carbon fiber plate increases longitudinal‐bending stiffness, improving RE by more than 3% [14]. This relatively stiff, curved plate shifts the ground reaction force anteriorly, generating a greater propulsive moment in late stance [15, 16] and reducing energy losses at the metatarsophalangeal joint (MTP joint [17]). Severing the plate medio‐laterally in the forefoot and midfoot regions to reduce longitudinal bending stiffness does not affect energetic cost [18]. However, RE is 1.87% lower in the Vaporfly 4% shoe compared to a prototype without the plate, suggesting that the plate provides benefits beyond longitudinal bending stiffness [12]. Removing either the carbon plate or replacing the PEBA foam with EVA foam increases energy expenditure, suggesting that both features contribute to the reduction in metabolic cost of running compared to controls [12]. The interaction and combination of both the plate and resilient foam may function as a distributed elastic structure [18, 19] but as yet, this idea remains untested.

The energetic benefits of AFT running shoes during level running are also observed during gradient running, though the benefits are less pronounced. Whiting et al. [20] demonstrated a reduction in metabolic power of 2.8% in athletes running in AFT on a 5% uphill gradient compared to a control. Similarly to level running, reducing the longitudinal bending stiffness during incline running had no effect on running energetics, although it did alter lower‐limb biomechanics, such as increasing negative metatarsophalangeal joint work and increasing positive ankle work [21]. During level running at a constant speed, each step involves the cyclic exchange of mechanical energy, where energy absorbed during the initial half of stance is equal to the positive work done during the latter half. As a result, there is no net change in the body's overall mechanical energy [22, 23, 24]. Theoretically all mechanical energy lost in the first half of stance could be stored elastically and returned in the second half of stance. The total energy turnover in each stance phase is approximately 100 J [25], and this cyclical exchange of energy occurs in biological structures in the limb (e.g., tendons in the distal limb such as the Achilles tendon and ligaments in the foot arch [25, 26], running shoe midsoles [3] and compliant running surfaces [27]). In level running, 43% of the overall energy exchange is via elastic structures, with the remainder of the energy required being generated by the skeletal muscles [28]. When running up an incline, the energy decrease in the first half of stance is less than the subsequent increase in positive mechanical energy that must be generated to raise the body's centre of mass and increase its potential energy [28, 29]. The energy that is stored elastically only decreases slightly at steeper gradients, however, because the positive energy increases dramatically with gradient, the stored elastic energy becomes a significantly smaller proportion of the total energy required [28, 29]. For example, at a 9° (15.8%) incline the elastic energy only accounts for 24% of the total mechanical energy required [28]. This reduced proportional contribution of elastic mechanisms during incline running means that net positive muscular work must be done by metabolically expensive concentric contractions [22], increasing the metabolic cost compared to level running. If the carbon plate and resilient midsole foam of AFT act together to confer elastic properties, the reduction in the relative contribution of elastic energy storage to the total mechanical energy change during incline running is expected to reduce the energetic benefit of wearing such shoes. The reduced metabolic energy expenditure benefit when running in the Vaporfly shoe compared to a control shoe on an incline [20] supports this hypothesis.

The aim of this investigation was to investigate the effects of incline on running energetics in a shoe incorporating AFT (the Nike ZoomX Vaporfly Next% shoe), characterized by a compliant PEBA foam midsole and curved carbon fiber plate, compared to a control running shoe with a similar PEBA foam midsole but lacking a midsole carbon fiber plate (the Nike ZoomX Streakfly shoe). It was hypothesized that the energetic benefit would decrease with increasing incline when running uphill.

## 2 Materials and Methods

### 2.1 Participants

Twelve male competitive runners capable of running 5 km in less than 18 min at the time of testing (age = 21.4 ± 1.2 years; mass = 70.5 ± 9.0 kg; height = 181.3 ± 7.1 cm; 5 k 16:22 ± 1:07 mm:ss) took part in the experiment. Sample size was determined a priori to ensure sufficient power to detect a 2% difference in RE while allowing for potential data exclusion. All participants volunteered and provided informed written consent to the experimental procedures, which were approved by the Faculty of Biological Sciences Research Ethics Committee at the University of Leeds (BIOSCI 19‐011 and FBS FREC 2024 2254‐2809) and complied with the declaration of Helsinki. Prior to the trials, participants were asked to maintain their usual diet and activity level whilst refraining from drinking alcohol in the preceding 24 h and avoid food and caffeine intake for at least 2 h before testing.

### 2.2 Running Shoes

**FIGURE 1:**
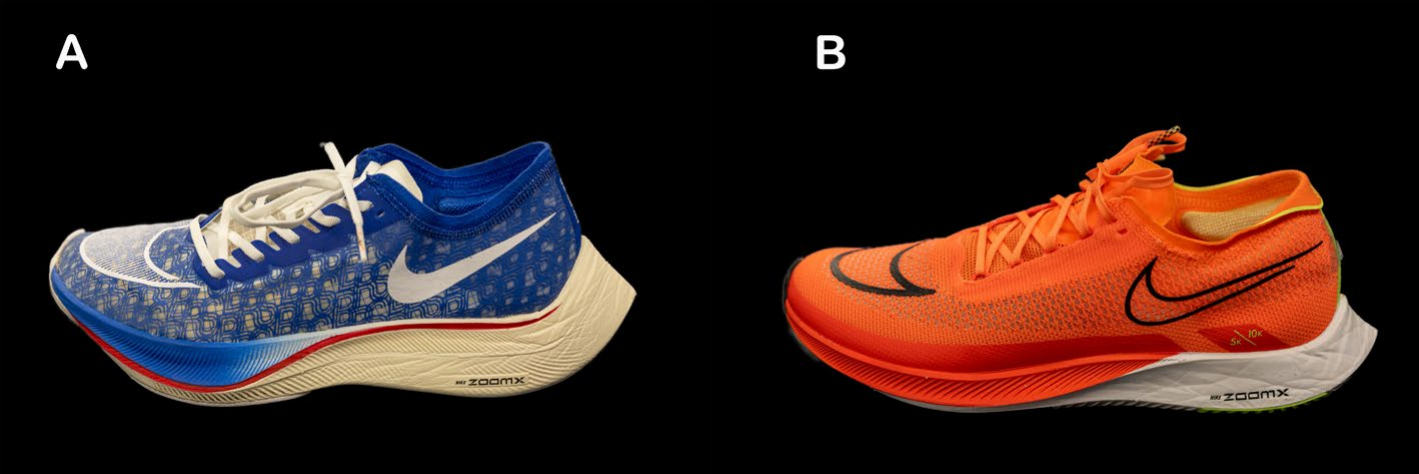
(A) Nike ZoomX Vaporfly Next% shoe (VF); (B) Nike ZoomX Streakfly shoe (SF).

Two running shoes were compared (Figure 1): the Nike ZoomX Vaporfly Next% shoe (VF) and the Nike ZoomX Streakfly shoe (SF). The VF incorporates a curved, midsole carbon fiber plate and ZoomX PEBA foam. The mass of the shoes is 200 g (UK size 9) and the stack height is 37.3 and 31.1 mm measured below the calcaneus and metatarsophalangeal joint, respectively (shoe unloaded). The SF incorporates a curved, midsole flexible plastic (Pebax) plate and ZoomX PEBA foam. The mass of the shoes is 187 g (UK size 9) and the stack heights below the calcaneus and metatarsophalangeal joint are 33.6 and 25.1 mm, respectively (shoe unloaded). Over the range of shoe sizes used, on average, the SF were 6.4% lighter than the VF in the corresponding size.

### 2.3 Experimental Protocol

Participants ran in each of the shoe conditions at a speed of 12 km h^−1^ at inclines of 0, 3%, 6% and 9% on a treadmill (HP Cosmos Pulsar, h/p/cosmos sports and medical gmbh, Nussdorf, Germany or PowerJog J100, Sport Engineering Ltd., Birmingham, UK). Each participant was tested across all conditions using the same treadmill. A running speed of 12 km h^−1^ was selected based off pilot testing at 9% incline, which confirmed that participants meeting the running performance inclusion criteria could sustain running aerobically at all tested gradients (i.e., maintaining a respiratory exchange ratio ≤ 1). Each participant completed a single trial at each combination of shoe and gradient. The shoe conditions were presented in a randomized order, and the incline order was also randomized within each shoe condition. Trials lasted 4–5 min until steady state *V̇*_O2_ and *V̇*_CO2_ were achieved. Between trials participants stood at rest until *V̇*_O2_ and *V̇*_CO2_ returned to baseline values.

### 2.4 Metabolic Measurements

A portable gas analysis system (METAMAX 3B, Cortex Biophysik GmbH, Leipzig, Germany or K5, COSMED Srl, Milan, Italy) was used to measure the submaximal rates of oxygen consumption (*V̇*_O2_) and carbon dioxide production (*V̇*_CO2_). Each participant was tested across all conditions using the same system.

Participants warmed up on the treadmill in their own running shoes at a self‐selected pace for approximately 5–10 min. Running economy (RE) was quantified as steady‐state mass‐specific *V̇*_O2_ (mL O_2_ kg^−1^ min^−1^) during submaximal running. Values were calculated from the final 60 to 90 s of each trial once a plateau in *V̇*_O2_ and *V̇*_CO2_ was achieved (typically ~5 min). Real‐time respiratory exchange ratio (RER; the ratio of *V̇*_CO2_ to *V̇*_O2_) was monitored throughout testing to ensure submaximal exercise (i.e., RER ≤ 1.0). Data for one participant at 9% incline were excluded due to their RER exceeding 1.0.

*V̇*_O2_ and *V̇*_CO2_ were normalized to body mass and are presented as gross metabolic rates.

### 2.5 Material Properties of Running Shoes

The mechanical (viscoelastic) properties of the running shoes were determined using an indentation test and a cantilever bending test.

#### 2.5.1 Indentation Test

Each intact shoe was loaded at the midfoot centred on the first metatarsophalangeal joint via a 36 mm circular steel plate indenter attached to an actuator (ElectroPuls E3000, Instron, High Wycombe, UK; dynamic accuracy 0.01 N). Cyclical loads (maximum load ~500 N at 4 Hz to simulate a loading period of ~185 ms^3^, and producing a maximum indentation of ~11 mm and a peak pressure of 49.1 N cm^2^, which is comparable to the peak pressures in the midsole [30]) were applied for 1001 cycles at room temperature (~20°C). Displacement and force during loading and unloading were sampled across repeated cycles at 200 Hz. For each cycle, energy stored and returned were calculated from force‐displacement loops and resilience calculated (resilience, % = energy returned/energy stored × 100). Mechanical properties were calculated from cycles 500 to 1000 where force‐displacement behavior had stabilized following pre‐conditioning. As the acquisition system recorded cycles at discrete intervals, analyses were performed on cycles 500, 600, 700, 800, 900 and 1000.

#### 2.5.2 Cantilever Bending Test

The longitudinal bending stiffness of the shoes was determined using a cantilever bending test. The shoe was fixed from the heel to the first metatarsophalangeal joint and a cyclical load applied at the forefoot tip, to produce a displacement of 23 mm, resulting in an angle of ~16° between the forefoot and hind foot. Displacement and force during loading and unloading were sampled across repeated cycles at 200 Hz. Longitudinal bending stiffness calculated from the average slope of the loading region of the force‐displacement loops between 20% and 80% of peak load after removing the load offset. Mechanical properties were calculated from cycles 500 to 1000, as described above.

### 2.6 Statistical Analysis

*V̇*_O2_ (mL O_2_ kg^−1^ min^−1^) was analyzed using a linear mixed‐effects model (LMM), with shoe condition, gradient and their interaction (shoe × gradient) as fixed effects and participant included as a random intercept to account for repeated measures. The interaction term was used to assess whether the relationship between *V̇*_O2_ and gradient differed between shoes. Where a significant shoe × gradient interaction was present, pairwise comparisons between shoe conditions at each gradient were performed using estimated marginal means with Bonferroni‐adjusted *p*‐values.

RE benefit of the VF compared to the SF was calculated as (*V̇*_O2,SF_ − *V̇*_O2,VF_)/*V̇*_O2,SF_. The relationship between RE benefit and gradient was characterized using an exponential model of the form RE benefit = *ae* ^*b*.Gradient^ fitted using non‐linear least squares. To statistically assess the effect of gradient on RE benefit, a LMM with gradient as a fixed effect and participant as a random intercept was applied.

All statistical analyses were carried out in R (version 4.5.3; R Core Team, 2025) using the *lme4*, *lmerTest*, and *emmeans* packages.

## 3 Results

### 3.1 Running Energetics

**FIGURE 2:**
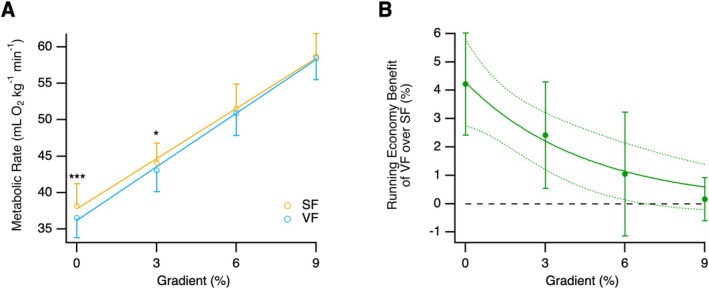
(A) Gross metabolic rate (*V̇*_O2_) of running in the Nike Vaporfly shoe (VF, blue) and the Nike Streakfly shoe (SF, orange) at four gradients: level, 3%, 6%, and 9%. Data are mean ± standard deviation. Linear regressions between metabolic rate and gradient are shown for each shoe condition. Statistically significant pairwise comparisons between shoe condition at a given gradient are indicated by *p < 0.05 and ***p < 0.001. (B) Running economy (RE) benefit of the VF compared to the SF at four gradients. Data are mean ± 95% confidence interval. The solid line shows the exponential fit (RE benefit = 4.28 *e* ^−0.22.Gradient^) with dotted lines indicating the 95% confidence limits. The dashed line represents zero RE benefit.

The relationship between *V̇*_O2_ and gradient was linear across the tested range (Figure 2A). A LMM demonstrated that there was a significant main effect of shoe (*F*(1,79) = 16.5, *p* < 0.001) and gradient (*F*(1,79) = 4574.9, *p* < 0.001), as well as a significant shoe × gradient interaction (*F*(1,79) = 4.8, *p* = 0.032). *V̇*_O2_ increased with gradient in both shoes, but the rate of increase was greater in the VF (2.48 ± 0.05 mL O_2_ kg^−1^ min^−1^%^−1^) than in the SF (2.32 ± 0.05 mL O_2_ kg^−1^ min^−1^%^−1^; *p* = 0.032). This resulted in convergence of *V̇*_O2_ at steeper gradients (Figure 2A). Post hoc comparisons (Bonferroni‐adjusted) indicated that *V̇*_O2_ was significantly lower in the VF compared to the SF during level running (∆ = 1.64 mL O_2_ kg^−1^ min^−1^, *p* < 0.001) and at 3% incline (∆ = 1.07 mL O_2_ kg^−1^ min^−1^, *p* = 0.018), with no significant difference at 6% or 9% incline (*p* ≥ 0.19).

RE benefit of VF decreased markedly with increasing gradient (Figure 2B). A LMM confirmed a significant negative effect of gradient on RE benefit (−0.42% ± 0.09% per % gradient; *t*_34_ = −4.69, *p* < 0.001). The relationship between RE benefit of the VF and gradient was also described by an exponential model (of the form RE benefit = *a e* ^*b*.Gradient^; *a* = 4.28 ± 0.76, *p* < 0.001; *b* = −0.221 ± 0.082, *p* = 0.0099; Figure 2B), explaining 23% of the variation in RE benefit (*R* ^2^ = 0.23). Based on this fitted model, predicted RE benefit was 4.22% during level running, decreasing by approximately 20% per 1% increase in gradient, to 2.42% at 3% incline, 1.05% at 1% incline and 0.52% on a 9% gradient.

### 3.2 Shoe Material Properties

**FIGURE 3:**
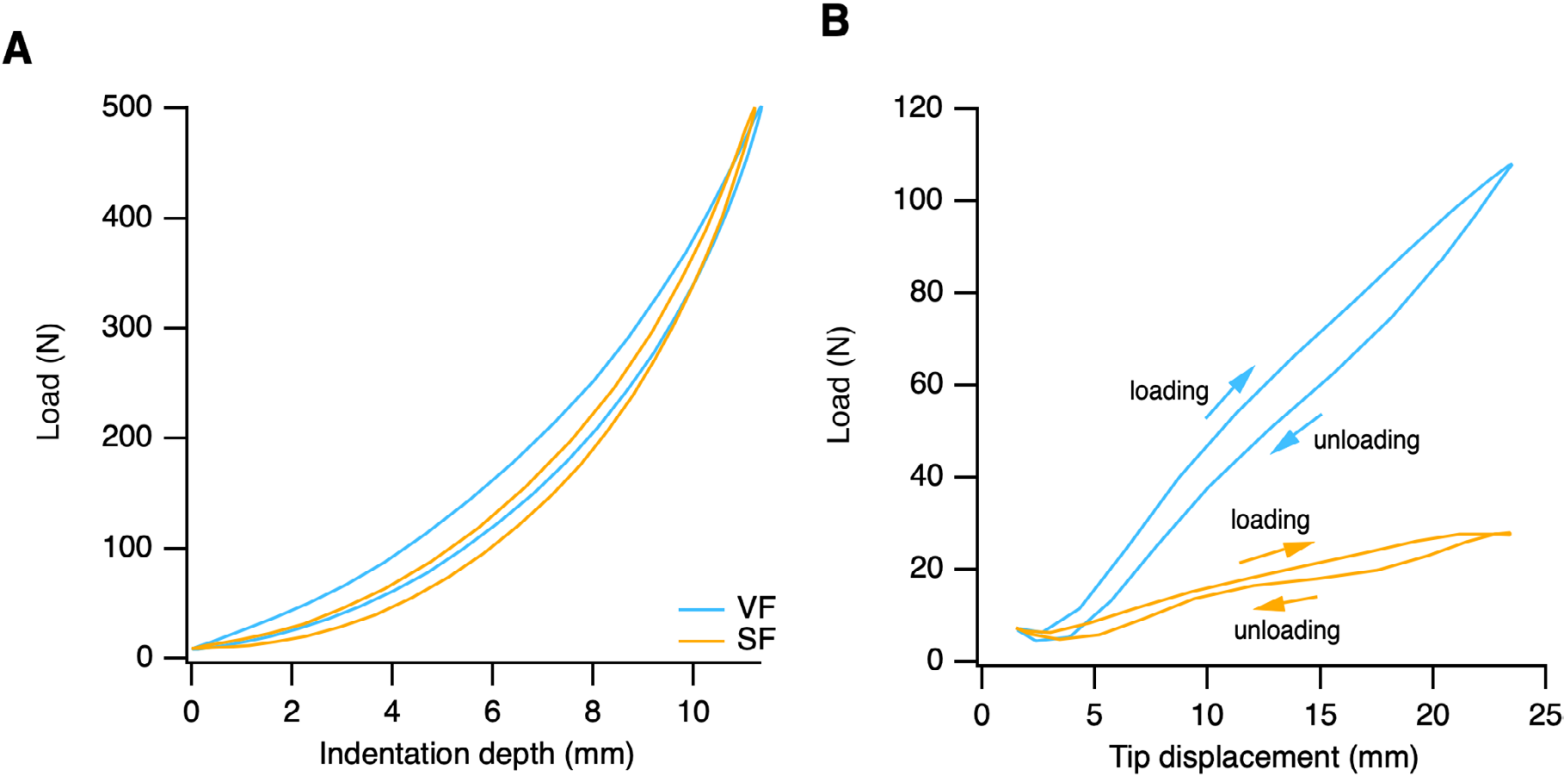
Representative load–displacement plots from (A) indentation tests and (B) cantilever bending tests in the Nike Vaporfly shoe (VF, blue) and Nike Streakfly shoe (SF, orange). Data are shown for a representative steady‐state cycle (cycle 1000). Load–displacement loops are oriented clockwise. Arrows in panel B indicate the direction of loading and unloading; arrows are omitted from panel A for clarity due to overlapping loops.

The material properties of the shoes are summarized in Figure 3 and Table 1. The VF stored more energy under compression than the SF but returned a smaller proportion of this energy during recoil—that is, had a lower resilience. The VF exhibited a ~4‐fold higher midfoot longitudinal bending stiffness compared to the SF. In bending, the VF was 4.26× stiffer than the SF and the SF stored and returned approximately 30% of the energy of the VF; the bending resilience was similar (Table 1).

**TABLE 1:** Material properties of the Nike Vaporfly shoe (VF) and the Streakfly shoe (SF) determined using indentation and cantilever bending tests.

Shoe	Indentation test				Cantilever bending test			
	Stored energy (J)	Returned energy (J)	Energy loss (J)	Resilience (%)	Bending stiffness (N/mm)	Stored energy (J)	Returned energy (J)	Resilience (%)
VF	2.07 ± 0.03	1.72 ± 0.02	0.35 ± 0.01	83.0 ± 0.28	4.99 ± 0.00	1.30 ± 0.01	1.12 ± 0.01	86.0 ± 0.24
SF	1.78 ± 0.02	1.55 ± 0.01	0.23 ± 0.01	87.3 ± 0.46	1.17 ± 0.01	0.41 ± 0.01	0.36 ± 0.01	86.0 ± 0.16

*Note:* Data are mean ± standard deviation calculated from cycles 500 to 1000. These cycles were selected to represent steady‐state behavior following pre‐conditioning. Standard deviations reflect cycle‐to‐cycle variability within a single test and therefore represent technical variability rather than between sample variation.

## 4 Discussion

This investigation demonstrated that when running on level and low inclines (3%) the VF reduce metabolic rate and confer a RE benefit compared to the SF. These differences likely reflect the greater longitudinal bending stiffness of the VF, due to its carbon fiber plate, together with an increased capacity for energy storage and return arising from its thicker PEBA midsole. However, the RE benefit decreases with increasing incline such that there is no reduction in metabolic rate or significant improvement in RE benefit on steeper inclines (6% and 9%). During level running, the VF provide a RE benefit of 4.2% relative to SF, which is in accordance with previous research: the VF shoes were found to improve RE by ~4% compared to a control shoe [3, 20, 31]. Although the running speed used in our study (12 km h^−1^) was slower than in previous research [3, 20], a similar RE benefit during level running was observed suggesting that the improvements in RE are independent of speed. This interpretation is consistent with findings from Paradisis et al. [8], although other work has reported lower benefits at slower speeds [31]. The energetic benefit of the VF (present study [3, 4, 5, 6]) has been shown to come from both the midsole carbon fiber plate and (PEBA) foam [12], which may operate together to function as a distributed elastic structure [18, 19]. Although the VF showed a slightly lower hysteresis (% energy recovery) during compressive loading than the SF, it stored and returned more energy (in both compression and bending), presumably due to the thicker midsole (~one‐third thicker), which is expected to reduce muscular work during running [22, 26]. This is also consistent with the reported reduction in gastrocnemius medialis muscle activity during the push‐off phase of stance in VF compared to a traditional racing shoe [32]. The reduction in both recruited muscle volume and muscular work is expected to reduce the overall metabolic energy expenditure of running [22].

The reduced RE benefit of the VF relative to the SF at steeper gradients is also consistent with previous findings. For example, Whiting et al. [20] reported a 2.8% reduction in metabolic rate during 3° (5.2%) incline running when using the VF, compared to a control shoe. Based on an exponential model of the relationship between RE benefit and gradient (Figure 2), our data suggest a slightly lower RE benefit of 1.4% when running on a 5.2% incline. In contrast, Hunter et al. [33] found no significant effect of gradient (4% decline, level, and 4% incline) on RE benefit when using a different AFT running shoe (Saucony Endorphin Pro), with an average RE benefit of 1.5% compared to a non‐mass matched control. However, direct comparison is limited because Hunter et al. [33] varied running speed with gradient and used a different running shoe.

The observed reduction in RE benefit with increasing incline supports our hypothesis that the smaller relative contribution of elastic energy storage and return to the total mechanical energy requirements of incline running [28], reduces the advantage of wearing AFT running shoes such as the VF. During uphill running, a greater proportion of mechanical work is required to raise the centre of mass, reducing the relative contribution of elastic mechanisms. In addition, reduced impact and braking forces likely limit the potential for elastic energy storage in compliant midsole foams, further diminishing the effectiveness of AFT.

In parallel with these changes in whole‐body energetics, runners adopt a more forefoot strike pattern during incline running [33, 34], which alters distal joint mechanics. It has been suggested that the energetic benefits of AFT may be greater for rearfoot strikers than for midfoot or forefoot strikers [3]. AFT is proposed to improve RE in level running by shifting the centre of pressure anteriorly and modifying lever mechanics, thereby increasing the effective ankle moment arm and reducing energy losses associated with MTP joint dorsiflexion [17, 35]. In addition, the curved geometry of the plate has been proposed to act as a lever, further enhancing ankle mechanical advantage during late stance [15]. However, a shift towards forefoot striking during uphill running may alter centre of pressure progression and reduce the effectiveness of these lever‐based mechanisms. Furthermore, forefoot striking may already reduce MTP joint energy losses due to reduced braking and normal impact forces [36], thereby diminishing the potential energy savings from increased longitudinal bending stiffness. Consistent with this, increasing longitudinal bending stiffness has been shown to increase metabolic energy expenditure when running on a 10% incline [37]. Similarly, Corbí‐Santamaría et al. [38] reported that AFT did not improve mountain running performance or indirect proxies of metabolic energy expenditure compared to a conventional mountain running shoe, reinforcing the limited benefit of AFT in uphill running. At gradients of 6% and 9% there was no detectable RE benefit of wearing the VF compared with the SF. Whilst the VF may still confer some energetic advantage over the SF on steeper inclines (as indicated by the significant exponential relationship), the magnitude of this effect is very small (e.g., ~0.5% at 9% incline) and is therefore unlikely to translate into a meaningful performance benefit.

We did not measure the effect of decline gradient on running energetics in the present study; however, previous work has reported a 2.4% reduction in metabolic power with the VF compared to a control during running on a 3° (5.2%) decline. Although relative RE benefits may be similar between incline and decline running [20], the absolute energetic cost of downhill running on shallow gradients is far lower than that of uphill running on an equivalent incline [29, 39] due to the reduced cost of eccentric compared with concentric muscle contractions [22]. Therefore, in the context of an undulating course, the absolute energetic benefit of AFT is likely to be dominated by sections involving level and shallow uphill running.

## 5 Limitations

The two shoes were not weight matched with the SF being ~6.4% lighter (~10–13 g per shoe) than the VF. Given that shoe mass is known to affect RE with a 1% increase in gross metabolic rate per 100 g added mass per shoe [7, 10] this small difference is expected to result in a small under‐estimation of RE benefit of the VF (0.06% at 9% incline to 0.1% on level) in comparison to the SF, which is unlikely to affect our conclusions. All participants were male as our convenience sample and inclusion criteria only yielded male runners. It is therefore unclear whether the decline in RE benefit with incline generalizes to female runners. However, as the proposed mechanisms relate to the elastic properties of the shoes and fundamental aspects of the biomechanics of incline running, our conclusions are likely to be broadly applicable. Participant familiarity with AFT was not recorded and may influence running biomechanics and energetics. However, previous studies have demonstrated improvements in RE with AFT with limited familiarisation [3, 6, 18, 21], suggesting that any influence of prior experience is unlikely to have substantially affected the present findings.

Although two treadmills and gas analysis systems were used, each participant was tested across all conditions on the same treadmill and with the same metabolic system. All comparisons were therefore made within‐participant, using the same equipment, minimizing potential bias due to inter‐device differences. Foot strike pattern was not controlled and may affect the energetic benefits of AFT. However, allowing participants to run naturally enhances the real‐world validity of the findings. While indentation and cantilever bending tests provide insight into running shoe material properties, they do not fully capture dynamic performance indicators such as rebound energy, coefficient of restitution, and potential energy deficit, which are critical in running performance [40]. Although the tests aimed to replicate appropriate strain rates, they remain limited in assessing energetic behavior because they impose a fixed deformation path and lack the inertial mass for realistic rebound.

## 6 Conclusions

The RE benefit of the VF relative to the SF decreased exponentially with gradient (*p* < 0.001; *R* ^2^ = 0.23) from 4.22% to 2.42% at 3% incline, 1.05% at 6% incline, and 0.52% at 9% incline. This demonstrates that the energetic advantage of AFT running shoes is progressively reduced as gradient increases. The reduced energetic cost of level and shallow incline running in the VF is likely attributable to the combined effects of increased longitudinal bending stiffness from the carbon fiber plate and greater energy storage and return from the thicker PEBA midsole, compared with shoes lacking a carbon plate and with a thinner PEBA midsole. However, the reduced relative contribution of elastic mechanisms with increasing incline, together with a greater reliance on net positive work generation, likely reduces the energetic benefit of such plated shoes at steep inclines.

Over an undulating course, AFT running shoes may still confer an overall energetic and therefore performance advantage, but this is likely to be driven primarily by their benefits during level running and on shallow inclines, where elastic energy storage and return contribute more substantially to locomotor energetics. Consequently, the magnitude of any performance benefit will depend on the proportion of time spent at lower versus higher gradients and may be reduced on courses characterized by sustained steep inclines.

## 7 Perspectives

Most major running shoe manufacturers now produce lightweight road racing models that incorporate a curved carbon fiber plate embedded within a compliant, resilient mid‐sole foam. The present findings have practical implications for competitive runners competing on undulating courses. Over variable terrain, the metabolic savings conferred by AFT footwear are likely to be greatest on level ground and shallow inclines, indicating that performance benefits may vary across different sections of a course rather than being uniformly distributed. Consequently, runners may derive greater energetic savings and potential tactical advantages on flat sections and gentle climbs, whereas on sustained steep gradients, where the contribution of elastic mechanisms is reduced, the performance gains from AFT footwear may be diminished.

## Author Contributions

G.N.A.: conceptualisation, methodology, investigation, formal analysis, supervision, writing original draft, writing review and editing. S.H.: investigation, formal analysis, writing review and editing. M.P.: investigation, formal analysis, writing review and editing. T.D.S.: conceptualisation, methodology, investigation, formal analysis, writing review and editing. D.C.: investigation (preliminary study that informed current hypothesis). A.P.B.: conceptualisation, methodology, investigation, formal analysis, supervision, writing original draft, writing review and editing.

## Funding

Funded by the University of Leeds.

## Conflicts of Interest

The authors declare no conflicts of interest.

## Data Availability

The datasets generated during and/or analyzed during the current study are available in the University of Leeds repository at https://doi.org/10.5518/1823.
